# Supplementary material for: Self-medication practice and contributing factors among pregnant women
Source: PLoS One. 2021 May 20;16(5):e0251725. doi: 10.1371/journal.pone.0251725 (PMC8136661; doi:10.1371/journal.pone.0251725)
Supplement: S1 Table — (DOC) [file pone.0251725.s001.doc]

**Data collection tool**

**Mekelle University**

**College of health science**

**School of pharmacy**

**Annex 1 : English version Questionnaire**

**Section 1: Socio demographic background of respondents**

1. Age (in Years) ______
2. Residence
   1. Rural
   2. Urban
3. Marital status
4. Single
5. Married
6. Divorced
7. Widowed
8. Others (specify)___________________
9. Religion
10. Orthodox
11. Muslim
12. Protestant
13. Catholic
14. Others (specify)_________________
15. Ethnicity
16. Tigraway
17. Amhara
18. Afar
19. Others (specify)__________________
20. Educational Status
    1. No formal education
    2. Primary school
    3. Secondary school
    4. Higher education
21. Occupation

a. Civil servant

b. Merchant

c. House wife

d. Others (specify)

1. Estimated monthly income in Ethiopian birr-----------------------
2. Alcohol 1. yes 2. no
3. Khat 1. yes 2. no
4. Smoking 1. yes 2. No
5. Chronic illness 1. yes 2. no
6. If yes what type………………..
7. Health insurance 1. yes 2. no

**Section 2: Obstetric history**

1. Gestational age
   1. First trimester
   2. Second trimester
   3. Third trimester
2. Order of the current pregnancy(gravidity)________
3. First
4. Second
5. Third and above
6. Number of children alive ______________
7. Number of Stillbirths________________
8. Have you ever had Antenatal follow up for the previous pregnancy?
9. Yes
10. No
11. If no why?
12. I don’t know about ANC
13. Negligence
14. It is not available
15. Others (specify)___________
16. Where do you delivered your last baby?
17. At home
18. Governmental health institution
19. Private clinic(hospital)
20. Other(specify)______________________
21. Have you ever faced complications related to previous pregnancy?
22. Yes
23. No
24. If yes, which one of the following?
25. vaginal bleeding
26. Sudden gush of fluid from vagina before onset of labor
27. Severe headache not relieved by simple analgesics,
28. Dizziness and blurring of vision
29. Excessive vomiting,
30. Swelling (hands, face, etc.),
31. Loss of fetal movements
32. Premature onset of contractions (before 37 weeks),
33. Severe or unusual abdominal pain
34. Others (specify)______________________

**Section 3: self-medication practice related questions**

1. Do use any medicine by yourself without consulting your physician?
   1. Yes
   2. No
2. If yes what kind of medicine ------------
3. Do you use any traditional medicine?
   1. Yes
   2. No
4. If yes what kind of TDM…………………
5. History of previous medication
   1. Yes
   2. No
6. From where do you find the medications
7. Pharmacy/drug stores
8. Leftover medicine
9. Sharing with family ,friends or neighbors
10. Others (specify)
11. How many times did you treat yourself with self-medications in the past one year? _____
12. What was (were) your reason(s) of self-medication? (Check more than one if applicable)

A. Cost saving B. ease of access medicines (Convenience) C. Poor health care service D. timesaving E. disease not series F. Others (specify)

1. For which of the following complaint(s) did you use self-medications? (Check more than one if applicable)

A. Nausea and vomiting B. headache C. Cold and Cough D. Sore throat E. Fever F.Allergic rhinitis H. Diarrhea J. Others (specify)

**Annex 2: Tigrigna Version Questionnaire**

**ሓበሬታ መእክቢ ቅፅ**

**አብ መቐለ ዩነቨርስቲ ጥዕና ኮሌጅ ፋርማሲ ት/ቢት**

**ቀዳማይ ክፋል - ማሕበራዊ ኩነት ተሓታቲ**

1. ዕድመ________________
2. መኖሬያ ሀ) ክተማ ለ) ገጠር
3. ኩነታት ሓዳር ______ ሀ) ዘይተመርዐወት ለ) ዝተመርዐወት ሐ) ዝተፋተሐት

መ) ስብአያ ዝሞታ ሰ) ካሊእ (ይገለጽ) ___________________

1. ሃይማኖት ሀ) ኦርተዶክስ ለ) ሙስሊም ሐ) ፕሮቴስታንት መ) ካቶሊክ

ሰ) ካሊእ (ይገለጽ)________

1. ብሄር ሀ) ትግራዋይ ለ) አማራ ሐ) ኦሮሞ መ) ካሊእ (ይገለጽ)________
2. ደረጃ ትምህርታ

ሀ) ዘይተምሃረት

ለ) ምጽሓፍን ምንባብን ዝኸአለት

ሐ) ቀዳማይ ደረጃ ትምህርቲ ዝተመሃረት

መ) ካልአይ ደረጃ ትምህርቲ ዝተመሃረት

ሰ) ኮሌጅ ዝተምሃረት

1. ኩነታት ስራሕ

ሀ) መዓልታዊ ስራሕ ለ) ሰራሕተኛ መንግስቲ ሐ) ነጋዳይ መ) ባዓልቲ ሓዳር

ሰ) ካሊእ (ይገለጽ) ___________________

1. ወርሓዊ አታዊ ____________________________
2. አልኮል መስተ ትሰትዪዶ ሀ) እወ ለ) አይሰትን
3. ጫት ትቕሕሚዶ ሀ) እወ ለ) አይቅሕምን
4. ሽጋራ ተትክኺዶ ሀ) እወ ለ) አየትክኽን
5. ሕዱር ሕማም አለኪዶ ሀ) እወ ለ) የብለይን
6. እወ እንተኾይኑ እንታይ ዓይነት ሕማም __________________
7. መድሕን ጥዕና አለኪዶ ሀ) እወ ለ) የብለይን

**ካልአይ ክፋል : ምሰ ወሊድ ዝተታ**ሕዘ **ታሪክ**

1. መበል ክንደይ ወርሒ እዩ ጥንስኺ

ሀ) ትሕቲ ሰለስተ ወርሒ ለ) ካብ ሰለስተ-ሽድሽተ ወርሒ ሐ) ልዕሊሽድሽተ ወርሒ

1. መበል ክንደይ ጥንስኺ እዩ ________

ሀ) ናመጀመርያ ለ) ካልአይ ሐ) ሳልሳይን ልዕሊኡን

1. ብሂወት ዘለዉ በዝሒ ቆልዑ ______________
2. ዝወረደ ጥንሲ ________________
3. አብቶም ዝሓለፉ ጥንስታት ቅድመ ወሊድ ክትትል ገይርክዶ ነይርኪ?

ሀ) እወ ለ) አይገበርኩን

1. አይገበርኩን እንተኾይኑ ንምንታይ?

ሀ) ብዛዕባ ቅድመ ወሊድ ክትትል አፍልጦ የብለይን ለ) ስለዘይተገደስኩሉ

ሐ) አብ ከባቢና ስለዘየለ መ) ካሊእ (ይገለጽ) ___________________

1. እቲ ናይ መጨረሻ ውላድኪ አበይ ወሊድኪ ?

ሀ) አብ ገዛ ለ) አብ ናይ መንግስቲ ትካል ጥዕና ሐ) አብ ናይ ዉልቀ ትካል ጥዕና መ) ካሊእ (ይገለጽ) _______________

1. አብ ሕሉፍ ጥንስኺ ጸገም ገጢሙኪዶ ነይሩ?

ሀ) እወ ለ) አይገጠመንን

1. እወ እንተኾይኑ እንታይ ዓይነት ፀገም ?

ሀ) ናይ ማህፀን ደም ምፍሳስ ለ) ድንገት ካብ ማህፀን ዝወፅእ ፈሳሲ ነገር ድሕሪ ወሊድ ሐ) ብቐሊል ፈውሲ ቓንዛ ዘይዕገስ ሕማም ርእሲ

መ) ድብርትን ብዥታን ሰ) ብዙሕ ተምላስ ረ) ሕብጠት (ኢድ፣ ፊት፣ወዘተ), ቀ) እቲ ዕሸል ምንቅስቓስ ጠጠው ምባል

በ) እዋኑ ዘይመልእ ወሊድ (ቅድሚ 37 ሰሙን), ተ) ሓያልን ዘይልሙድን ሕማም ከብዲ ቸ) ካሊእ (ይገለጽ) ___________________

**ሳልሳይ ክፋል : ኩነታት ምጥቃም መድሓኒት**

1. ሓኪም ተይአማከረኪ ባዕልኪ መድሓኒተ ትውሰዲ ዶ

ሀ) እወ ለ) አይተጠቐምኩን

1. እወ እንተኾይኑ ታይ ዓይነት ________________________________
2. ባህላዊ መድሓኒት ተጠቒምኪዶ ትፈልጢ?

ሀ) እወ ለ) አይተጠቐምኩን

1. እወ እንተኾይኑ ታይ ዓይነት ________________________________
2. ናይ ቅደሚ ሕዚ ዓርሰ ሕክምና ታሪክ

ሀ) እወ ለ) አይተጠቐምኩን

1. ንዓርሰ ሕከምና ዝኮነ መድሓኒተ ካበይ ትረክቢ ?

ሀ) አብ መደብር መድሓኒት ለ) ቅድም ሕዚ ተአዚዞምለይ ካብዝተረፉ መድሓኒታት ሐ) ምስ ቢተሰብ/ዓርኪ/ጎረቢት በምክፋለ መ)ካሊእ (ይገለጽ) ___________________

1. ብዘይ ናይ በዓል ሞያ ምኽሪ አብ ዝሓለፈ ሓደ ዓመት ክንደይ ጊዘ ዓርሰ ሕካምና ገይርኪ ? _____
2. ዓርሰ ሕካምና ክትገብሪ ዝገበረኪ ምኽንያታት እንታይ እዮም? (ካብ ሓደ ብላዕሊ ምኽባብ ይካአልዩ)

ሀ) ወፃኢ ንምቕናስ ለ) ምችው ስለዝኾነ ሐ) ዝዉሃብ ሕክምና ድካማ ሰለዝኮነ መ) ግዘ ንመቑጣብ ሠ) ሕማመ ቀሊል ሰለዝኮነ ረ ) ካሊእ (ይገለጽ) ___________________

1. ነይኖም ሕማመት ዓርሰ ሕካምና ገይርኪ ? (ካብ ሓደ ብላዕሊ ምኽባብ ይካአልዩ)

ሀ) ንዕዉልዉልን ተምላስን ለ) ሕማም ርእሲ ሐ) ሰዓል መ) ጉረሮ ምቑሳል ሠ) ሙቐት ሰውነት ረ) ምሕኻኽን ቃንዛን ቀ) ውፅአት በ) ካሊእ (ይገለጽ) _____________

***ንትሕብብርኩም የመስግን***
