# Supplementary material for: Self-medication practice and contributing factors among pregnant women
Source: PLoS One. 2021 May 20;16(5):e0251725. doi: 10.1371/journal.pone.0251725 (PMC8136661; doi:10.1371/journal.pone.0251725)
Supplement: Attachment — Submitted filename: PONE-D-20-32462_reviewer_GARE.pdf [file pone.0251725.s003.pdf]

PLOS ONE

Self-medication practice and contributing factors among pregnant women

--Manuscript Draft--

**Manuscript Number:**	PONE-D-20-32462
**Article Type:**	Research Article
**Full Title:**	Self-medication practice and contributing factors among pregnant women
**Short Title:**	self-medication practice during pregnancy
**Corresponding Author:**	Yirga Legesse Niriayo, Msc<br>Mekelle University College of Health Sciences<br>Mekelle, Tigray ETHIOPIA
**Keywords:**	Pregnancy; self-medication; Ethiopia
**Abstract:**	Background<br><br>The practice of self-medication during pregnancy is a global challenge that necessitates high attention as it poses a potential threat to the pregnant mother and fetus. However, little known regarding self-medication practice and its contributors among pregnant mothers in our setting.<br><br>Objective<br><br>The main aim of this study was to investigate the practice of self-medication and its contributing factors among pregnant women<br><br>M<br><br>eth odology: A cross sectional study was conducted among pregnant women at antenatal care follow-up of Ayder comprehensive specialized hospital, Tigray, Ethiopia. Written informed consent was obtained from each participant before interview. Simple random sampling technique was employed to recruit participants in to the study. Data were collected by interviewing participants using the structured questionnaire. Binary logistic regressions analysis was performed to determine the contributing factors of self-medication practice during pregnancy. A p value of less than 0.05 was considered as significant.<br><br>Results<br><br>A total of 250 pregnant women were included in the study. Of the total, 40.8% practiced self-medication during the current pregnancy. Morning sickness (39.2%), headache (34.3%), and upper respiratory tract infections (29.4%) were the leading indications for self-medication. According to participant report, ease of access to medicines (25.5%), feelings that the disease is minor (21.6%) and timesaving (19.6%) were the most commonly reported reasons for self-medication practice. Absence of health insurance (AOR: 2.75, 95%CI: 1.29-5.89) and being on first trimester of pregnancy (AOR: 2.44, 95%CI: 1.02-5.86) were significant contributors of self-medication practice among pregnant women.<br><br>Conclusion<br><br>In our study, high prevalence of self-medication was reported among pregnant women. Self-medication practice during pregnancy was higher among pregnant women on first trimester and those who were not having health insurance. Therefore, intervention programs should be designed to minimize the practice of self-medication during pregnancy.
**Order of Authors:**	Yirga Legesse Niriayo, Msc
	Kadra Mohammed
	Solomon Weldegebreal Asgedom

Powered by Editorial Manager® and ProduXion Manager® from Aries Systems Corporation

<table>
<tr><td rowspan="3"></td><td>Gebre Teklemariam Demoz</td></tr>
<tr><td>Shishay Wahdey</td></tr>
<tr><td>Kidu Gidey</td></tr>
<tr><td>Additional Information:</td><td></td></tr>
<tr><td>Question</td><td>Response</td></tr>
<tr><td>Financial Disclosure

Enter a financial disclosure statement that describes the sources of funding for the work included in this submission. Review the submission guidelines for detailed requirements. View published research articles from PLOS ONE for specific examples.

This statement is required for submission and will appear in the published article if the submission is accepted. Please make sure it is accurate.

Unfunded studies
Enter: The author(s) received no specific funding for this work.

Funded studies
Enter a statement with the following details:
• Initials of the authors who received each award
• Grant numbers awarded to each author
• The full name of each funder
• URL of each funder website
• Did the sponsors or funders play any role in the study design, data collection and analysis, decision to publish, or preparation of the manuscript?
• NO - Include this sentence at the end of your statement: The funders had no role in study design, data collection and analysis, decision to publish, or preparation of the manuscript.
• YES - Specify the role(s) played.

* typeset</td><td>The author(s) received no specific funding for this work.</td></tr>
<tr><td>Competing Interests

Use the instructions below to enter a competing interest statement for this submission. On behalf of all authors, disclose any competing interests that could be perceived to bias this</td><td>The authors have declared that no competing interests exist</td></tr>
</table>

work—acknowledging all financial support and any other relevant financial or non-financial competing interests.<br><br>This statement **will appear in the published article** if the submission is accepted. Please make sure it is accurate. View published research articles from *PLOS ONE* for specific examples.<br><br>**NO authors have competing interests**<br><br>Enter: *The authors have declared that no competing interests exist.*<br><br>**Authors with competing interests**<br><br>Enter competing interest details beginning with this statement:<br><br>*I have read the journal's policy and the authors of this manuscript have the following competing interests: [insert competing interests here]*<br><br>* typeset	
**Ethics Statement**<br><br>Enter an ethics statement for this submission. This statement is required if the study involved:<br><br>• Human participants<br>• Human specimens or tissue<br>• Vertebrate animals or cephalopods<br>• Vertebrate embryos or tissues<br>• Field research<br><br>Write "N/A" if the submission does not require an ethics statement.<br><br>General guidance is provided below. Consult the submission guidelines for detailed instructions. **Make sure that all information entered here is included in the Methods section of the manuscript.**	This study was approved by ethics review committee of school of pharmacy, College of Health Sciences, Mekelle University. Each study participant was well informed about the objective of the study. After getting permission from the ACSH hospital, written informed consent was obtained from all participants. Confidentiality was assured for all the information provided. All the methods were performed in accordance with approved institutional guidelines.

**Format for specific study types**<br><br>**Human Subject Research (involving human participants and/or tissue)**<br>• Give the name of the institutional review board or ethics committee that approved the study<br>• Include the approval number and/or a statement indicating approval of this research<br>• Indicate the form of consent obtained (written/oral) or the reason that consent was not obtained (e.g. the data were analyzed anonymously)<br><br>**Animal Research (involving vertebrate animals, embryos or tissues)**<br>• Provide the name of the Institutional Animal Care and Use Committee (IACUC) or other relevant ethics board that reviewed the study protocol, and indicate whether they approved this research or granted a formal waiver of ethical approval<br>• Include an approval number if one was obtained<br>• If the study involved *non-human primates*, add *additional details* about animal welfare and steps taken to ameliorate suffering<br>• If anesthesia, euthanasia, or any kind of animal sacrifice is part of the study, include briefly which substances and/or methods were applied<br><br>**Field Research**<br><br>Include the following details if this study involves the collection of plant, animal, or other materials from a natural setting:<br>• Field permit number<br>• Name of the institution or relevant body that granted permission	
**Data Availability**<br><br>Authors are required to make all data underlying the findings described fully available, without restriction, and from the time of publication. PLOS allows rare exceptions to address legal and ethical concerns. See the PLOS Data Policy and FAQ for detailed information.	Yes - all data are fully available without restriction

A Data Availability Statement describing where the data can be found is required at submission. Your answers to this question constitute the Data Availability Statement and **will be published in the article**, if accepted.<br><br>**Important:** Stating 'data available on request from the author' is not sufficient. If your data are only available upon request, select 'No' for the first question and explain your exceptional situation in the text box.<br><br>Do the authors confirm that all data underlying the findings described in their manuscript are fully available without restriction?	
**Describe where the data may be found in full sentences. If you are copying our sample text, replace any instances of XXX with the appropriate details.**<br><br>• If the data are **held or will be held in a public repository**, include URLs, accession numbers or DOIs. If this information will only be available after acceptance, indicate this by ticking the box below. For example: *All XXX files are available from the XXX database (accession number(s) XXX, XXX.).*<br>• If the data are all contained **within the manuscript and/or Supporting Information files**, enter the following: *All relevant data are within the manuscript and its Supporting Information files.*<br>• If neither of these applies but you are able to provide **details of access elsewhere**, with or without limitations, please do so. For example:<br><br>*Data cannot be shared publicly because of [XXX]. Data are available from the XXX Institutional Data Access / Ethics Committee (contact via XXX) for researchers who meet the criteria for access to confidential data.*<br><br>*The data underlying the results presented in the study are available from (include the name of the third party*	All relevant data are within the manuscript and its Supporting Information files.

and contact information or URL).<br>• This text is appropriate if the data are owned by a third party and authors do not have permission to share the data.<br><br>* typeset	
Additional data availability information:	

Powered by Editorial Manager® and ProduXion Manager® from Aries Systems Corporation

manuscript

Click here to access/download;Manuscript;self-self medication for plose final.doc

Self-medication practice and contributing factors among pregnant women

Yirga Legesse Niriayo*[1], Kadra Mohammed[1], Solomon Weldegebreal Asgedom[1], Gebre Teklemariam Demoz[2], Shishay Wahdey[3] Kidu Gidey [1],

Author's address:

[1] Department of Clinical Pharmacy, School of Pharmacy, College of Health Sciences, Mekelle University, Mekelle, Tigray, Ethiopia.

[2] Clinical Pharmacy and Pharmacy Practice Unit, Departments of Pharmacy, College of Health Sciences, Aksum University, Axum, Tigray, Ethiopia.

[3] Department of Public reproductive health, School of public health , College of Health Sciences, Mekelle University, Mekelle, Tigray, Ethiopia

*Corresponding author: Yirga L., E–mail: yirga.legesse@mu.edu.et OR yirga.pharma@gmail.com , Tel.: +251-914-86-18-82

Abstract

Background: The practice of self-medication during pregnancy is a global challenge that necessitates high attention as it poses a potential threat to the pregnant mother and fetus. However, little known regarding self-medication practice and its contributors among pregnant mothers in our setting. **Objective:** The main aim of this study was to investigate the practice of self-medication and its contributing factors among pregnant women

Methodology: A cross sectional study was conducted among pregnant women at antenatal care follow-up of Ayder comprehensive specialized hospital, Tigray, Ethiopia. Written informed consent was obtained from each participant before interview. Simple random sampling technique was employed to recruit participants in to the study. Data were collected by interviewing participants using the structured questionnaire. Binary logistic regressions analysis was performed to determine the contributing factors of self-medication practice during pregnancy. A p value of less than 0.05 was considered as significant.

Results: A total of 250 pregnant women were included in the study. Of the total, 40.8% practiced self-medication during the current pregnancy. Morning sickness (39.2%), headache (34.3%), and upper respiratory tract infections (29.4%) were the leading indications for self-medication. According to participant report, ease of access to medicines (25.5%), feelings that the disease is minor (21.6%) and timesaving (19.6%) were the most commonly reported reasons for self-medication practice. Absence of health insurance (AOR: 2.75, 95%CI: 1.29-5.89) and being on first trimester of pregnancy (AOR: 2.44, 95%CI: 1.02-5.86) were significant contributors of self-medication practice among pregnant women.

Conclusion: In our study, high prevalence of self-medication was reported among pregnant women. Self-medication practice during pregnancy was higher among pregnant women on first trimester and those who were not having health insurance. Therefore, intervention programs should be designed to minimize the practice of self-medication during pregnancy.

Key words: Pregnancy, self-medication, Ethiopia

Introduction

According to the World Health Organization (WHO), self-medication is defined as the act of using medications by patients or individuals to treat self-diagnosed disorders or symptoms on their own initiative without getting advice from health care provider [1-3] . Self- medication includes use of over counter drugs available without a physician's prescription, irregular use of prescribed medicines, use of leftover drugs from previous prescriptions, and use of herbal and traditional medicines [4, 5]. Owing to the limited access to health care service and scarcity of resources, the practice of self-medication is a common in developing countries including Ethiopia [6, 7] . According to the meta-analysis reported in 2018, the prevalence of self-medication in Ethiopia ranged from 12.5%-78.1% with an average of 44%[8].

The practice of self-medication during pregnancy has been increasing worldwide, particularly in developing countries owing to the poor health care system [9-11]. Likewise, the practice of self-medication during pregnancy is increasing in Ethiopia with a reported prevalence ranged from 15.5%-70% [8, 12, 13].

In clinical practice, self-medication during pregnancy still remained a significant medical challenge [14, 15]. Although drugs are frequently used during pregnancy in clinical practice, their safety has not been well-established as pregnant women are often excluded from clinical trials due to the fear of harm on the mother or the developing fetus [15, 16]. Therefore, unless absolutely necessary, drugs should be avoided during pregnancy[17]. Despite this fact, there is high level of self-medication use during pregnancy[18]. Self-medication can affect both the fetus and the mother and it could cause detrimental effects on the fetus including malformation/teratogenicity, fetal toxicity, low birth weight, premature birth, respiratory problems as well as death [15, 18, 19]. It has been reported that at

least 10% of birth defects are resulted from the exposure of pregnant women to drugs [15] .

The prevalence of self-medication varies across different communities and it could be affected by several factors including lack of access to healthcare service, unregulated distribution of medicines, patients' attitudes toward healthcare providers, socio-economic factors, long waiting times, cost of the drugs, educational level, age, income, education level, satisfaction, and belief of people's toward medication and disease.[9, 12, 20-22]

Despite the potential harmful effect of self-mediation practice during pregnancy [15, 16], there is little awareness about the impact of self-medication practice among pregnant mothers in developing countries including Ethiopia [7, 10, 23]. Hence, evaluation of self-medication practice and its contributors will provide information for health policy makers and relevant stakeholders to develop strategies to prevent the risks associated with self-medication practice during pregnancy. In our setting, though there are some studies on self-medication in general population [7, 8], studies regarding self-medication practices during pregnancy are scanty. Our study, therefore, investigated the practice of self-medication and its contributing factors during pregnancy.

Methodology

Study design and setting

An institutional based cross-sectional study was conducted from January to April 2019 at antenatal care follow up of Ayder comprehensive specialized hospital (ACSH), Tigray region, Ethiopia. ACSH is the largest public hospital in Tigray region whiles it the second in Ethiopia next to black lion hospital. It provides service for about 10 million people in the catchment area.

Study participants and data collection procedure

All pregnant mothers who had antenatal care (ANC) follow up in ACSH were the source population. Pregnant women at any gestational age who came for ANC to ACSH hospital during the study period were included in this study. Pregnant women who are critically ill to give response, unable to hear/ communicate and those who are unwilling to give consent were excluded from the study.

The sample size for this study was determined using the single population proportion formula for the prevalence of self-medication practice. Accordingly a sample of 262 participants was calculated assuming 26.6% prevalence of self-medication practice during pregnancy according to a study conducted in Addis Abeba [24], 95% confidence level, 5% margin of error, and 10% contingency for nonresponse rate. From 262 participants approached, 12 patients were excluded from the study due to unable to hear [1], critically ill to give response [4], and unwillingness to give consent [7]. The participants were recruited into the study during their appointment for ANC using simple random sampling technique

We collected the data using an interviewer administered structured questionnaire (S1 Table). . The questionnaire was developed based on previous studies [9, 13, 14] and it was amended to fit the current study. The questionnaire was translated to local language (Tigrigna), and then back translated to English to ensure consistency of meaning. Pre-test was carried out on 5% of the sample before the commencement of the actual data collection and slight amendments were made on the questionnaire based on the findings. The questionnaire involves data related to socio-demographics, obstetrics factor, and self-medication practices. Fifth year clerkship pharmacy students were employed to collect the data for this study and they were given training and orientation.

Data analysis

We analyzed the data using the Statistical Package for the Social Science (SPSS version 24.0) (S2 Table). Descriptive statics was computed as frequency, mean and standard deviation (SD). Multicollinearity was checked to test correlation among predictor variables using variance inflation factor (VIF) and none was collinear. The association of each independent variable with self-medication practice was determined using univariable logistic regression analysis. Furthermore, the variables with p value <0.25 in univariable analysis were re-analyzed using multivariable binary logistic regression model to identify the independent predictors of self-medication practice during pregnancy

Ethics

This study was approved by ethics review committee of school of pharmacy, College of Health Sciences, Mekelle University. Each study participant was well informed about the objective of the study. After getting permission from the ACSH hospital, written informed consent was obtained from all participants. Confidentiality was assured for all the information provided. All the methods were performed in accordance with approved institutional guidelines.

Result

Socio-demographic characteristics

A total of 250 participants were included in this study. The mean (±SD) age was 26.9±5.42. Most (83.6%) of the participants have attended primary school and above. Nearly half (46.4%) were housewives. Majority of the participants were from urban. The mean income was 4543.42±3436.23 Ethiopian Birr. Alcohol consumption was reported in 17.2% of the participants (Table 1).

Table 1. **Socio-demographic characteristics of participants (n=250)**

Category	Frequency (%)
**Age**	
≤18	2(0.8)
19-25	115(46)
26-30	83(33)
31-35	34(13.6)
>35	16(6.4)
**Residence**	
Urban	183(73.2)
Rural	67(26.8)
**Educational status**	
No formal education	36(14)
Primary	41(16.4)
Secondary	108(43.2)
Higher education	65(26)
**Occupation**	
civil servant	59(23.6)
Merchant	50(20)
Housewife	116(46.4)
Others	25(10)
**Income**	
<5000	126(50.4)
>=500	124(49.6)
**Chronic illness**	
Yes	11(4.4)
No	239(95.6)
**Alcohol**	
No	207(82.8)
Yes	43(17.2)

Obstetric factors

Nearly half (44.8%) of the participants were in the first trimester of their pregnancy and majority (66%) were multigravidas. About two-thirds (62.8%) of participants had ANC follow-up in their previous pregnancy and 16.8% experienced complications related to previous pregnancy (Table 2).

Table 2: Obstetrics characteristics of study participants, 2019(n=250)

Category	Frequency (%)
**Gestational period**	
First trimester	112(44.8)
Second trimester	101(40.4)
Third trimester	37(14.8)
**Gravidity**	
Primeravida	85(34)
Mutigravida	165(66%)
**Previous ANC follow up**	
Yes	157(62.8)
No	93(37.2)
**Place of delivery of last baby**	
Home	8(4.8)
Health institution	157(93.2)
Previous still birth	
No	227(90.8)
Yes	23(9.2)
**Previous pregnancy related complications**	
Yes	42(16.8)
No	208(83.2)

Self-medication practice during pregnancy

Of the total, 40.8% practiced self-medication during the current pregnancy while one-fourth (25.2%) of participants had previous self-medication experience. Among those who used self-medication (102), 43(42.2%) medicated themselves with modern medicine and 41(40.2%) used traditional medicine while 18(17.6%) used both modern medicine and traditional medicine. The participants mentioned deferent reasons for self-medication. Among those, the major reasons for self-medication were easily accessing medicines (25.5%), feeling that the disease is minor (21.6%), and timesaving (19.6%). Morning sickness (39.2%), headache (34.3), and upper respiratory tract infections (29.4%) were the most common indications for self- medication.

Table 3. Self-medication practice during pregnancy, 2019(n=250)

Characteristics	Frequency (%)
**Self-medication**	
Yes	102(40.8)
No	148(59.2)
**History of previous self-medication**	
Yes	63(25.2)
No	187(74.8)
**Type of medicine used**	
Modern only	43(42.2)
TDM only	41(40.2)
Both modern and TDM	18(17.6)
**Reason for self-medication**	
Easily accessing medicines	26(25.5)
Disease not serious	22(21.6)
timesaving	20(19.6)
Poor health service provision	15(14.7)
cost saving	13(12.7)
Lack of trust on prescribers	6(5.9)
**Common Indications for self-medication**	
Morning sickness	40(39.2)
Headache	35(34.3)
Upper respiratory tract infections	30(29.4)
Dyspepsia	22(21.6)
Urinary tract infections	20(19.6)
cough and cold	16(15.7)
Diarrhea	14(13.7)
Allergic rhinitis	8(7.8)
**Source of modern medication for self-medication**	
Pharmacies/drug stores	60(24)
Leftover medicine	32(12.8)
Sharing with family ,friends or neighbors	10(4)

Factors associated with self-medication practice during pregnancy

Univariable logistic regression analysis was performed to compare pregnant women with self-medication and without self-medication using the socio-demographic and obstetrics related characteristics. Accordingly, gestational age (COR:2.90,95%CI:1.25-6.70), health insurance (COR:3.06,95% CI:1.63-5.74), gravidity (COR:1.79, 95%CI:1.03-3.10) were significantly associated in with self-medication practice in univariate analysis. Moreover, variables with P<0.25 in the univariable analyses were re-analyzed using multivariable logistic regression model. The whole model containing all predictors was statistically significant (Chi-square = 27.676, df = 10, P=0.002). In multivariable logistic regression analysis, participants without health insurance (AOR: 2.75, 95%CI: 1.29-5.89) and participants on first trimester (AOR: 2.44, 95%CI: 1.02-5.86) were more likely to practice self-medication compared to those with health insurance and on third trimester, respectively (Table 4).

Table 4. Factors associated with self-medication practice during pregnancy (n=250)

Characteristics	Self-medication		COR (95% CI)	p-value	AOR (95% CI)	p-value
	No, n (%)	Yes, n (%)				
Residence						
Urban	113(76.4)	70(68.6)	1	1	1	1
Rural	35(23.6)	32(31.4)	1.48(0.84-2.60)	0.177	0.80(0.37-1.74)	0.577
Educational status						
No formal education	18(12.2)	18(17.)	1.41(0.62-3.19)	0.41	0.82(0.29-2.29)	0.702
Primary	17(11.5)	24(23.5)	1.99(0.90-4.30)	0.090	1.30(0.52-3.21)	0.574
Secondary	75(50.7)	33(32.4)	0.62(0.33-1.17)	0.143	0.60(0.30-1.15)	0.123
College and above	38(25.7)	27(26.5)	1	1	1	1
Chronic illness						
No	144(97.3)	95(93.1)	1	1	1	1
Yes	4(2.7)	7(6.9)	2.653(0.756-9.310)	0.128	1.228(0.237-6.36)	0.807
Health insurance						
Yes	20(13.5)	33(32.4)	1	1	1	1
No	128(86.5)	69(67.6)	3.061(1.634-5.735)	0.000	2.75(1.29-5.89)	0.009
Gravidity	58(39.2)	27(26.5)	(1)	1	1	1
Primigravida	90(60.8)	75(73.5)	1.228(0.64- 2.354)	0.537	0.63(0.34-1.18)	0.152
Multigravida	58(39.2)	27(26.5)	(1)	1	1	1
Previous pregnancy related complication						
No	128(86.5)	80(78.4)	1	1	1	1
Yes	20(13.5)	22(21.6)	2.669(1.202-5.926)	0.016	1.20(0.56-2.61)	0.637
Gestational age						
First trimester	58(39.2)	54(52.9)	2.99(1.25-6.69)	0.013	2.44(1.02-5.9)	0.045
Second trimester	62(41.9)	39(38.2)	1.96(0.84-4.58)	0.122	1.87(0.77-4.54)	0.166
Third trimester	28(18.9)	9(8.8)	1	1	1	1

Discussion

The practice of self-medication is a global challenge that necessitates high attention because it poses a potential threat to the pregnant mother and fetus [14, 25]. In developing countries including Ethiopia, clinicians may not be aware of the actual burden of self-medication and its contributing factors during pregnancy. Thus, conducting such kind of study will help them to design and develop strategies to prevent /minimize self-medication practice during pregnancy. Our study, therefore, investigated the practice of self-medication and its contributing factors among pregnant women. The current study revealed that a significant proportion of pregnant women practiced self-medication either with modern and/or herbal medicine without consulting healthcare professionals.

Despite the potential harmful effect of self-mediation during pregnancy [15, 16], nearly half (40.8%) of pregnant women practiced self-medication during their current pregnancy. This result is comparable with the findings reported from Tanzania [14] and Iran [22]. In contrast, higher findings were reported from previous studies conducted in Democratic Republic of Congo [26], Nigeria [27] and Ethiopia [12]. On the other hand, our finding is higher compared to the findings reported from Addis Abeba [24] and Netherland [28]. These variations could be attributed to the differences in level of awareness about risks of self-medication in pregnancy, population demographics, access to healthcare service, and restriction policies of dispensing practices.

Morning sickness, headache, and upper respiratory tract infections were the leading indications for self-medication in this study. In Tanzanian study [14], malaria, morning sickness, and headache were the leading illness that led to self-medication. Unlike our study, malaria was the most common indication for self-medication in Tanzanian study [14]. This could be due to the less prevalence of malaria in our study setting.

In the present study, the most commonly reported reasons for self-medication practice during pregnancy were ease of access to medicines, feeling that disease is minor and prolonged waiting time. In agreement with our study, similar finding were reported from previous studies conducted in Addis Ababa[24] and democratic republic of Congo[26]. The ease of access to medications without prescription could be attributed to the poor drug regulatory system and weak enforcement on restricting prescription drugs sale without prescription as well as nonprescription drugs sale to pregnant women. Moreover, it could be augmented due to the lack of attention and priorities of health policy makers and other stakeholders on the burden of self-medication risks[24]. Therefore, necessary measures should be taken to strengthen regulatory system and enforce regulations so as to reduce the practice of self-medication during pregnancy.

Our study revealed that pregnant women without health insurance were more prone to self-medication practice which is consistent with a finding reported from Iran [22]. The possible explanation is that those who have health insurance are more likely to visit health institution and see a doctor. Thus, they are more likely to get prescribed medication as the cost of their visit and medication can be compensated by the insurance.

Pregnant women on first trimester were more likely to practice self-medication compared to those on third trimester. This finding is supported by a study conducted in Tanzania [14]. The possible justification for higher self-medication practice during first trimester of pregnancy could be due to the more frequent occurrence of symptoms and/or illnesses including morning sickness, headache, and fever in the first trimester than other trimester during pregnancy. More importantly, this finding is worrisome as drug exposure in this period is more likely to cause congenital abnormalities [29-31]. Therefore, more emphasis should be given to the use of medication during first trimester of pregnancy.

Finally, our study had some limitations. Our study was a cross–sectional suggesting that it cannot provide adequate evidence of causality regarding self-medication and its contributing factors. During interview, pregnant women were expected to recall information from their past experience; therefore, recall bias might affect the study findings. The findings of our study could be affected by the difference in population demographics, healthcare system and regulations and knowledge of the participants. Therefore, it should be extrapolated to other countries with caution.

Conclusion

In our study, a high prevalence of self-medication was reported among pregnant women. Self-medication practice during pregnancy was higher among pregnant women on first trimester and those who were not having health insurance. Therefore, healthcare providers should provide more emphasis to the risky participants and implementation of national health insurance needs to be encouraged. Moreover, intervention programs should be designed to minimize the practice of self-medication during pregnancy.

Acknowledgments

We would like to acknowledge the data collectors and the hospital staff for their genuine cooperation. Our gratefulness goes to the pregnant women for their eager involvement in the study.

References

1. Rutter P. Role of community pharmacists in patients' self-care and self-medication. Integrated pharmacy research & practice. 2015;4:57.
2. Mahapatra T. Self-care and self-medication: A commentary. Annals of Tropical Medicine and Public Health. 2017;10(3):505.
3. Organization WH. Guidelines for the regulatory assessment of Medicinal Products for use in self-medication. World Health Organization, 2000.
4. Pfaffenbach G, Tourinho FS, Bucaretchi F. Self-medication among children and adolescents. Current drug safety. 2010;5(4):324-8.
5. Bennadi D. Self-medication: A current challenge. Journal of basic and clinical pharmacy. 2013;5(1):19.
6. Parulekar M, Mekoth N, Ramesh C, Parulekar A. Self medication in developing countries a systematic review. 2016.
7. Ayalew MB. Self-medication practice in Ethiopia: a systematic review. Patient preference and adherence. 2017;11:401-13. doi: 10.2147/PPA.S131496. PubMed PMID: 28280312.
8. Sisay M, Mengistu G, Edessa D. Epidemiology of self-medication in Ethiopia: a systematic review and meta-analysis of observational studies. BMC Pharmacol Toxicol. 2018;19(1):56-. doi: 10.1186/s40360-018-0248-8. PubMed PMID: 30201045.
9. Abeje G, Admasie C, Wasie B. Factors associated with self medication practice among pregnant mothers attending antenatal care at governmental health centers in Bahir Dar city administration, Northwest Ethiopia, a cross sectional study. The Pan African medical journal. 2015;20:276-. doi: 10.11604/pamj.2015.20.276.4243. PubMed PMID: 26161199.
10. Kureshee NI, Dhande PP. Awareness of Mothers and Doctors about Drug Utilization Pattern for Illnesses Encountered during Pregnancy. J Clin Diagn Res. 2013;7(11):2470-4. Epub 11/10. doi: 10.7860/JCDR/2013/6329.3582. PubMed PMID: 24392375.
11. Adanikin AI, Awoleke JO. Antenatal drug consumption: the burden of self-medication in a developing world setting. Tropical doctor. 2017;47(3):193-7.

12. Jambo A, Mengistu G, Sisay M, Amare F, Edessa D. Self-Medication and Contributing Factors Among Pregnant Women Attending Antenatal Care at Public Hospitals of Harar Town, Ethiopia. Front Pharmacol. 2018;9:1063-. doi: 10.3389/fphar.2018.01063. PubMed PMID: 30337871.
13. Zewdie T, Azale T, Shimeka A, Lakew AM. Self-medication during pregnancy and associated factors among pregnant women in Goba town, southeast Ethiopia: a community based cross sectional study. BMC research notes. 2018;11(1):713-. doi: 10.1186/s13104-018-3821-8. PubMed PMID: 30305180.
14. Marwa KJ, Njalika A, Ruganuza D, Katabalo D, Kamugisha E. Self-medication among pregnant women attending antenatal clinic at Makongoro health centre in Mwanza, Tanzania: a challenge to health systems. BMC Pregnancy Childbirth. 2018;18(1):16-. doi: 10.1186/s12884-017-1642-8. PubMed PMID: 29310609.
15. Nakamura MU, Kulay Junior L, Pasquale M. [Use of drugs during pregnancy: benefit and cost]. Revista brasileira de ginecologia e obstetricia : revista da Federacao Brasileira das Sociedades de Ginecologia e Obstetricia. 2008;30(1):1-4. Epub 2009/01/15. doi: 10.1590/s0100-72032008000100001. PubMed PMID: 19142535.
16. Araújo DD, Leal MM, Santos EJV, Leal LB. Consumption of medicines in high-risk pregnancy: evaluation of determinants related to the use of prescription drugs and self-medication. Brazilian Journal of Pharmaceutical Sciences. 2013;49(3):491-9.
17. Sachdeva P, Patel BG, Patel BK. Drug use in pregnancy; a point to ponder! Indian J Pharm Sci. 2009;71(1):1-7. doi: 10.4103/0250-474X.51941. PubMed PMID: 20177448.
18. Rahmani A, Hamanajm SA, Fallahi A, Ghanei Gheshlagh R, Dalvand S. Prevalence of self-medication among pregnant Women: A systematic review and meta-analysis. Nursing and Midwifery Studies. 2019;8(4):169-75.
19. Ciarkowski SL, Stalburg CM. Medication safety in obstetrics and gynecology. Clinical obstetrics and gynecology. 2010;53(3):482-99. Epub 2010/07/28. doi: 10.1097/GRF.0b013e3181ec14c0. PubMed PMID: 20661034.
20. Karimy M, Rezaee-Momtaz M, Tavousi M, Montazeri A, Araban M. Risk factors associated with self-medication among women in Iran.

BMC public health. 2019;19(1):1033-. doi: 10.1186/s12889-019-7302-3. PubMed PMID: 31370891.
21. Bello FA, Morhason-Bello IO, Olayemi O, Adekunle AO. Patterns and predictors of self-medication amongst antenatal clients in Ibadan, Nigeria. Niger Med J. 2011;52(3):153-7. doi: 10.4103/0300-1652.86124. PubMed PMID: 22083501.
22. Ebrahimi H, Atashsokhan G, Amanpour F, Hamidzadeh A. Self-medication and its risk factors among women before and during pregnancy. The Pan African medical journal. 2017;27:183-. doi: 10.11604/pamj.2017.27.183.10030. PubMed PMID: 28904710.
23. Nergard CS, Ho TPT, Diallo D, Ballo N, Paulsen BS, Nordeng H. Attitudes and use of medicinal plants during pregnancy among women at health care centers in three regions of Mali, West-Africa. J Ethnobiol Ethnomed. 2015;11:73-. doi: 10.1186/s13002-015-0057-8. PubMed PMID: 26453339.
24. Beyene KGM, Beza SW. Self-medication practice and associated factors among pregnant women in Addis Ababa, Ethiopia. Tropical medicine and health. 2018;46:10-. doi: 10.1186/s41182-018-0091-z. PubMed PMID: 29743807.
25. Adanikin AI, Awoleke JO. Antenatal drug consumption: the burden of self-medication in a developing world setting. Trop Doct. 2017;47(3):193-7. Epub 2016/06/16. doi: 10.1177/0049475516653067. PubMed PMID: 27302199.
26. Mbarambara PM, Songa PB, Wansubi LM, Mututa PM, Minga BBK, Bisangamo CK. Self-medication practice among pregnant women attending antenatal care at health centers in Bukavu, Eastern DR Congo. International Journal of Innovation and Applied Studies. 2016;16(1):38.
27. Abasiubong F, Bassey EA, Udobang JA, Akinbami OS, Udoh SB, Idung AU. Self-Medication: potential risks and hazards among pregnant women in Uyo, Nigeria. The Pan African medical journal. 2012;13:15-. Epub 09/19. PubMed PMID: 23308320.
28. Verstappen GM, Smolders EJ, Munster JM, Aarnoudse JG, Hak E. Prevalence and predictors of over-the-counter medication use among pregnant women: a cross-sectional study in the Netherlands. BMC public health. 2013;13:185-. doi: 10.1186/1471-2458-13-185. PubMed PMID: 23452432.

29. Ban L, West J, Gibson JE, Fiaschi L, Sokal R, Doyle P, et al. First trimester exposure to anxiolytic and hypnotic drugs and the risks of major congenital anomalies: a United Kingdom population-based cohort study. PLoS One. 2014;9(6):e100996.
30. Mashuda F, Zuechner A, Chalya PL, Kidenya BR, Manyama M. Pattern and factors associated with congenital anomalies among young infants admitted at Bugando medical centre, Mwanza, Tanzania. BMC research notes. 2014;7(1):1-7.
31. Dellicour S, Sevene E, McGready R, Tinto H, Mosha D, Manyando C, et al. First-trimester artemisinin derivatives and quinine treatments and the risk of adverse pregnancy outcomes in Africa and Asia: A meta-analysis of observational studies. PLoS Med. 2017;14(5):e1002290-e. doi: 10.1371/journal.pmed.1002290. PubMed PMID: 28463996.

Supporting information

S1 Table. Data collection instrument.
(DOCX)
S2 Table. Dataset.
(ZIP)

Click here to access/download
Supporting Information
data collection tool self.doc

Click here to access/download
Supporting Information - Compressed/ZIP File Archive
self medication SPSS data.sav
