# Supplementary material for: Self-medication practice and contributing factors among pregnant women
Source: PLoS One. 2021 May 20;16(5):e0251725. doi: 10.1371/journal.pone.0251725 (PMC8136661; doi:10.1371/journal.pone.0251725)
Supplement: Attachment — Submitted filename: response to reviewers for plose one.docx [file pone.0251725.s004.docx]

**Manuscript number:** PONE-D-20-32462

**Title: “Self-medication practice and contributing factors among pregnant women”**

**Authors:** Yirga Legesse Niriayo*^1^, Kadra Mohammed^1^**,** Solomon Weldegebreal Asgedom^1^, Gebre Teklemariam Demoz^2^, Shishay Wahdey^3^ Kidu Gidey ^1^,

**Authors’ response to academic editor’s and reviewers’ comments**

We thank the academic editor and the reviewer for reviewing our manuscript. We greatly appreciate the academic editor and reviewers for their constructive comments and suggestions	on our submitted manuscript. We have modified our manuscript based on the editor and reviewer comments and suggestions. We offer below responses to each of the points raised	by the academic editor and reviewers. We have also attached the modified manuscript with track changes and without track change based on the editor’s and reviewers’ comments and suggestions with our resubmission. Please note that all page and line numbers we have mentioned below refer to the resubmitted manuscript with track changes.

**Response to academic editor comments:**

**Journal requirements**

1. Please ensure that your manuscript meets PLOS ONE's style requirements, including those for file naming. The PLOS ONE style templates can be found at

**Response: We have ensured that all style requirements are addressed.**

2. For more information on PLOS ONE's expectations for statistical reporting, please see [https://journals.plos.org/plosone/s/submission-guidelines.#loc-statistical-reporting](https://www.google.com/url?q=https%3A%2F%2Fjournals.plos.org%2Fplosone%2Fs%2Fsubmission-guidelines.%23loc-statistical-reporting&sa=D&sntz=1&usg=AFQjCNFT0bKhTwuyMnanDf3fy0S1njsRZQ). Please update your Methods and Results sections accordingly

**Response:** **We thank you. We have done so with our resubmission.**

3. Please include additional information regarding the survey or questionnaire used in the study and ensure that you have provided sufficient details that others could replicate the analyses. For instance, if you developed a questionnaire as part of this study and it is not under a copyright more restrictive than CC-BY, please include a copy, in both the original language and English, as Supporting Information. If the original language is written in non-Latin characters, for example Amharic, Chinese, or Korean, please use a file format that ensures these characters are visible.

We have made all data, including data set and data collection tool fully available as supporting information.

**Response: We have included the data collection tool used in this study that contains both English and Tigrigna version as supporting information.**

4. We noticed you have some minor occurrence of overlapping text with the following previous publication(s), which needs to be addressed:

- https://tropmedhealth.biomedcentral.com/articles/10.1186/s41182-018-0091-z

In your revision ensure you cite all your sources (including your own works), and quote or rephrase any duplicated text outside the methods section. Further consideration is dependent on these concerns being addressed.

**Response: We have paraphrased some of the overlapping texts. Actually, the overlaps are occurred just by chance. Moreover, we have cited all utilized resources.**

**.**

**Response to Reviewer comments**

1. Overall, the text is well organized and structured according to IMRED. The writing style is pleasant. However, the presentation of tables does not meet current standards for presentation of a table. It will remove the left and right borders and remove the intermediate lines only the bottom line and the variable banner (the 2 top lines

**Response: We appreciate the reviewer comments. We have done so with our resumption. Please see the highlighted tables (Table 1, Table 2, Table 3, and Table 4)**

2. There are also shortcomings in the presentation of the references, in particular reference 17 to be corrected.

**Response: We thank the reviewer’s comments and suggestions. We have corrected them with our resubmission. Please see the highlighted references with yellow colour.**

3. Statistical analyses are carried out to high technical standards and are described in sufficient detail. However, we should have presented the results of the univariate analysis with the overall p value for a variable instead of the p value per variable category; especially for variables with more than 2 modalities.

**Response: Thank you for your suggestions. Actually, both approaches (overall p value for a variable and p value per variable category) are possible. We have modified it according to your suggestions in our resubmission. Please see the highlights on table 4.**

4. In the presentation of table 4, for the reference modality, the p value must not appear (in yellow in the table). The presentation of Table 4 needs to be improved

**Response: We appreciate the reviewer comments. We have done so with our resubmission. Please see the highlights on table 4.**

5. Make a clear distinction between the variables that were used for the univariate analysis and those that were retained for the multivariate analysis.

**Response: We have done so with our resubmission. Please see the highlights on page 10 lines 209-221.**
